# Changes in organic matter composition caused by EDTA washing of two soils contaminated with toxic metals

**DOI:** 10.1007/s11356-021-15406-z

**Published:** 2021-07-28

**Authors:** Erika Jez, Carlo Bravo, Domen Lestan, Simon Gluhar, Ladislau Martin-Neto, Maria De Nobili, Marco Contin

**Affiliations:** 1 Wine Research Centre, University of Nova Gorica, Vipavska cesta 13, 5000 Nova Gorica, Slovenia; 2 Department of Agricultural, Food, Environmental and Animal Sciences, University of Udine, Via delle Scienze 206, 33100 Udine, Italy; 3 Biotechnical Faculty, University of Ljubljana, Jamnikarjeva 101, 1000 Ljubljana, Slovenia; 4 Embrapa Instrumentação, CP 741, São Carlos, SP 13560-970 Brazil

**Keywords:** Potentially toxic metals, Soil washing, EDTA, Humic substances, Soil organic matter, Humic acids, Fulvic acids

## Abstract

Two soils contaminated with potentially toxic metals (PTMs) contrasting in pH and mineralogy were remediated with CaEDTA, and changes in soil organic matter (SOM) composition were investigated. Previous studies showed no significant loss of SOM from CaEDTA-treated soils, but the results of our study reflected significant decreases (from 46 to 49%) in the free fraction of humic acids (HAs). Remediation affected the composition of the free HA fraction via disturbance of intermolecular bonds — an increase in phenolic and aromatic groups with a simultaneous decrease in carbohydrates — which was confirmed by FTIR spectroscopy in both soils. Because non-radical molecules such as carbohydrates were selectively removed, the concentration of free radicals in the free HA fraction increased in acidic soil. The bound fraction of HAs and fulvic acids (FAs) in SOM, which are important due to their stability and the permanent effects they have on the soil’s physical properties, remained unchanged in both remediated soils. The effect of soil recultivation was observed only in the excitation emission matrix (EEM) fluorescence spectra of HAs. In terms of SOM, CaEDTA soil washing can be considered moderately conservative; however, the restoration of free humic fractions is likely to be a long-term process.

**Supplementary Information:**

The online version contains supplementary material available at 10.1007/s11356-021-15406-z.

## Introduction

Soils contaminated by potentially toxic metals (PTMs) and in particular by lead (Pb), zinc (Zn), and cadmium (Cd) are a widespread problem (Edogbo et al. 2020). Pb and Cd are the most toxic elements whose extensive use (ore mining, tailings, and smelting) has caused environmental and health problems in many parts of the world (Das et al. 2018; Doumas et al. 2018; Huang et al. 2018; Contin et al. 2019; Varrica et al. 2018;).

Various biological, chemical, and physical approaches are used for the elimination of PTMs from soils (Ashraf et al. 2017). If the concentration of PTMs in soils contaminated by anthropogenic activities is several times the threshold value for toxicity, soil washing can be an effective alternative to solidification/stabilization and landfilling (Liu et al. 2021). Several inorganic acids (e.g., hydrochloric, sulfuric, and nitric acid), organic acids (e.g., oxalic, citric, gluconic, and ascorbic acid), synthetic or biodegradable chelating agents (e.g., EDDS, EDTA, GLDA, NTA), and surfactants (e.g., rhamnolipids and sophorolipids) have been tested to improve the efficiency of soil washing (Gluhar et al. 2020; Peng et al. 2018). Among them, EDTA has proven to be one of the best, most environmentally friendly, and most economical (Gluhar et al. 2020; Jelusic et al. 2014; Jez and Lestan 2016; Lestan 2017). Although EDTA soil washing is an efficient and permanent way of removing PTMs from contaminated soils, it may affect soil properties (Gluhar et al. 2020). In fact, in addition to targeted PTMs, EDTA forms strong complexes with a variety of cations, including alkaline earth metals (Ca and Mg) and other major soil components such as Al and Fe. This often has a detrimental effect on the structure and physical properties of the soil matrix (Zeng et al. 2005). Begum et al. (2013) and Hartley et al. (2014) observed the dissolution of native Fe/Al (hydro)oxides and large amounts of Ca carbonates in soils treated with Na_2_EDTA (about 50% of the Ca was removed). To avoid this problem, Lestan (2017) developed a novel remediation method known as ReSoil® washing technology in which the chelating agent is present in its CaEDTA form (ethylenediaminetetraacetic acid calcium disodium salt (C_10_H_12_N_2_O_8_CaNa_2_)), which improves stability of soil aggregates (Zupanc et al. 2014), and consequently has a lower effect on soil fertility (Theodoratos et al. 2000).

Soil organic matter (SOM), generally measured as soil organic C (SOC), is the main indicator of soil quality and agronomic sustainability, as it affects the physical, chemical, and biological properties of soil. Many of these soil properties influence plant growth (Magdoff and Weil 2004). It is known that SOC decreases in long-term PTM-polluted soils (Viventsova et al. 2005), but soil washing of contaminated soils may also have negative effects on SOM. Many studies have reported that after soil washing with Na_2_EDTA, the SOM did not change (Zupanc et al. 2014; Tahmasbian et al. 2017; Hosseini et al. 2018). Sometimes, changes in SOC are difficult to investigate because these changes occur slowly and are relatively small compared to the vast SOC pool size, but some of the soil C fractions, such as humic acids, are more sensitive than the total SOC (Slepetiene et al. 2011). Although fulvic and humic acids (FAs and HAs) are distinct and remarkably uniform across soils, they do not exist per se in nature (Magdoff and Weil 2004). HAs and FAs are used as SOM proxies in soils even though 50% or more of the organic material in mineral soils resides in non-extractable humin (Rice 2001). Nevertheless, the humic substance-based approach for understanding the natural processes of SOM and plant growth has been widely adopted (Olk et al. 2019; Hayes and Swift 2020; De Nobili et al. 2020), and it can be a useful tool for characterizing the washing effect on soil. During washing treatment, intensive mixing of the soil slurry and soil compression after de-watering in particular (Lestan 2017) can lead to the decomposition of polymeric humic materials. Goulas et al. (2017) characterized the organic matter in the washing solution and found that the main organic compounds were FAs, HAs, glycosylated proteins, melanoidin, and lignocellulose. However, to our knowledge, detailed information on whether soil washing has a negative effect on HAs has not been reported. To gain better insights into the qualitative changes in HAs caused by soil washing, spectroscopic methods should be used. In fact, studying the composition and quality of SOM is often more important than just looking at quantitative changes (Zhou et al. 2018). Lu et al. (2018) proposed the use of a combination of UV–vis spectrometry, Fourier transform infrared (FTIR) spectroscopy, and fluorescence spectrometry to investigate HA composition.

We hypothesized that EDTA soil washing would change the composition of humic substances in treated soils by influencing the way they bind to soil minerals and their supramolecular aggregation and that soil pH and mineralogy would play a major role. The binding of FAs and HAs to clays and oxides is indeed dependent on the formation of cationic bridges with polycations such as Ca^2+^ (Bachmann et al. 2008; Rowley et al. 2018). In washed soils, Ca^2+^ is in excess because it is added in the form of CaEDTA (Lestan 2017). Mahieu et al. (2002) and Olk et al. (2006) showed by ^13^C-CPMAS-NMR that the Ca^2+^-bound fraction of HAs (extracted by the complexing action of pyrophosphate) corresponded to more humified materials. Conversely, the fraction that was free of polycationic binding to minerals and therefore directly extractable by sodium hydroxide was consistently composed of younger, more labile materials (De Nobili et al. 2008). These fractions were useful for assessing the chemical and biochemical stabilization of SOC in soils under different managements (Olk et al. 2019).

The soil properties after ReSoil® washing technology have been evaluated several times in previous studies (Lestan 2017; Gluhar et al. 2020; Kaurin et al. 2020) and have shown no significant or lasting effects, apart from a slight increase in the pH value of the soil due to the lime treatment of the process solutions. However, the qualitative effect of the remediation on SOM remains largely unknown. The focus of this study was to evaluate the qualitative changes induced by ReSoil® washing technology with CaEDTA on SOM. The changes in the humic (FA + HA) and non-humic (NH) fractions were quantified, taking into account their association with mineral soil components (free and bound) in two PTM-polluted soils with contrasting pH and mineralogy that had been subjected to soil washing. We completed our study by characterizing the extracted free and bound HAs using different spectroscopic techniques (UV-vis, excitation emission matrix (EEM), FTIR, electron spin resonance (ESR)) and tested whether two cultivation cycles were able to restore the original composition of the SOM.

## Materials and methods

### Soil sampling and analyses

The soils used in this experiment were collected from the upper 30-cm layer of an active farmland near Arnoldstein, Austria (acidic soil) and a vegetable garden in the Meza Valley, Slovenia (calcareous soil). Lead mining and smelting for more than 300 years in the Meza Valley and more than 500 years in Arnoldstein caused severe accumulation of Pb, Zn, and Cd in these soils. Soil PTMs were analyzed by flame (acetylene/air) atomic absorption spectrophotometry (FAAS) (Varian AA240FS) with deuterium background correction after microwave-assisted acid extraction (USEPA 3052, 1995).

The pH was measured in a 1/2.5 (w/v) suspension of soil and 0.01 M CaCl_2_. Carbonates were determined volumetrically following the method proposed by Kalra and Maynard (1991).

Total organic carbon (TOC) was determined by automated thermal analyses where carbon is converted to CO_2_ by flash combustion at 1080°C (Costech Elemental Combustion System 102 elemental analyzer (Costech Instruments) coupled with a Thermo Scientific 103 Delta V Advantage Isotope Ratio Mass Spectrometer (Thermo Scientific)). Carbonates were previously removed from 10 mg of soils by treatment with HCl in silver capsules.

### EDTA soil washing and recultivation of washed soils

Remediation of contaminated soils (ReSoil®) was carried out in the pilot-scale chelate washing plant described by Lestan (2017). Briefly, soil (40 kg per batch), at about 18% moisture content, was washed with recycled EDTA solution (50 L) for 18 h in a concrete mixer. The calcareous soil was washed with solutions containing 60 mmol kg^−1^ EDTA, whereas 100 mmol kg^−1^ EDTA was used for the acidic soil. Oversized materials (>2 mm) were separated from the contaminated soil by wet screening. The soil solid phase was separated from the washing solution using a chamber filter press. In a downstream section of the process, the washed soil was rinsed in a filter press with three rinsing solutions recycled from the previous batch and then with fresh water (15 L) to compensate for water losses (the difference in moisture between the soil entering and exiting the process). The untreated last rinsing solution from the previous batch was used for the first rinsing solution (50 L). Used washing solution (50 L), and the second (50 L) and third (50 L) rinsing solutions were treated (and the EDTA washing solution recycled as CaEDTA) by alkalization with lime (pH > 12). Solid wastes, the precipitate of toxic metals hydroxides and toxic metals absorbed on waste paper, were removed in a chamber filter press and by vacuum filtration. The remaining EDTA was recovered as insoluble H_4_EDTA from the acidic phase of the second rinsing solution (after the addition of 96% H_2_SO_4_, pH 2–2.3) by vacuum filtration. The recycled washing solution was prepared from EDTA recycled as CaEDTA and the addition of H_4_EDTA and fresh Na_4_EDTA to compensate for the lost chelant (10–15%). For each soil, the process was carried out three times and then the batches of the same soil were merged together. Remediated soil was passed through a 5-mm sieve to recreate the soil structure and stored at room temperature for 30 days.

Experimental plots (dimensions 70 × 40 cm, 30 cm high) filled with remediated soils were fertilized with MnSO_4_ as recommended by Jelusic et al. (2014) at a rate equivalent to 40 kg ha^−1^, right before planting a selection of vegetable species. The first series of crops planted in spring 2017 was spinach (*Spinacia oleracea*), parsley (*Petroselinum crispum*), and salad (*Lactuca sativa*), and the second series planted in autumn 2017 was spinach and Chinese cabbage (*Brassica rapa* Pekinensis). Sampling (1 kg of soil) was carried out in October when autumn crops were still present.

### SOC extraction and fractionation

**Fig. 1:**
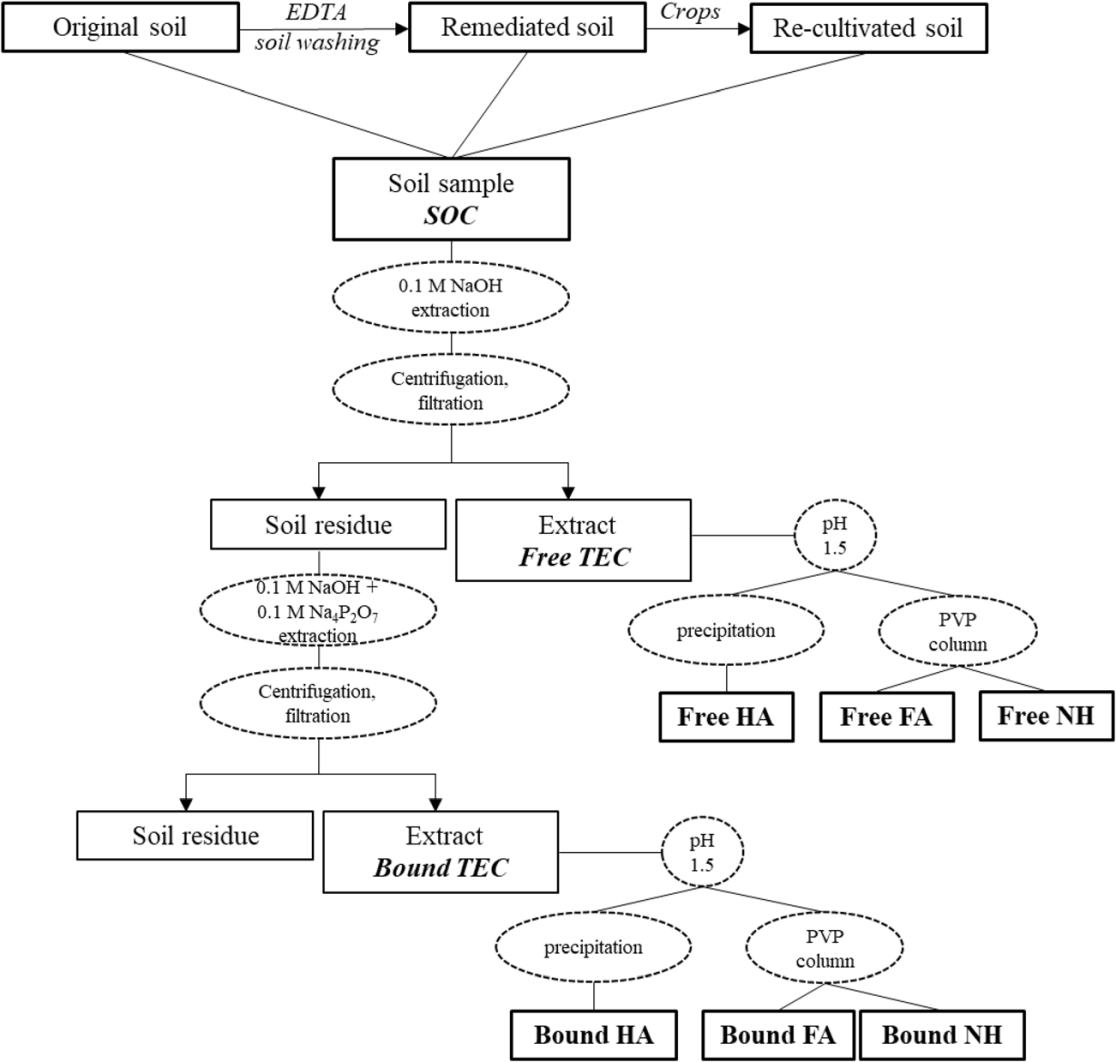
Layout of soil washing experiment and SOM extraction and fractionation procedures

Sequential extractions and fractionations were performed on air-dried soil samples according to the scheme reported in Fig. 1. Free and bound fractions were obtained by sequentially extracting 50 g of soil with 500 mL of 0.5 M NaOH (free total extractable C (TEC)) initially and then with 500 mL of 0.1 M Na_4_P_2_O_7_ plus 0.1 M NaOH (bound TEC). Both extractions were carried out under an N_2_ saturated atmosphere for 1 h. The soil-extractant suspensions were then centrifuged (20 min at 14,000 RPM) to separate extracted organic matter from soil mineral constituents and filtered through 0.2-μm cellulose filters. Free and bound HAs were obtained by precipitation at pH 1.5 (using 96% H_2_SO_4_) from the corresponding TEC extracts, separated by centrifugation, washed with acidified distilled water, and then freeze dried. The supernatant, containing FA and NH fractions, was fractionated by adsorption chromatography on cross-linked polyvinylpyrrolidone (PVP) columns according to the procedure described by De Nobili and Petrussi (1988). The NH fraction was leached from the PVP column and collected, while adsorbed FAs were eluted with 0.5 M NaOH.

For UV-vis and fluorescence spectroscopies, HA fractions were re-dissolved in 0.1 M phosphate buffer (pH 7.0).

Total extractable carbon (TEC) and organic C in the FA and NH fractions were measured with a total organic carbon analyzer (TOC-VCPN, Shimadzu) after appropriate dilution and neutralization of solutions.

### Humic acid characterization

#### C/N ratio and carbon isotope composition

The content of carbon (%C) and nitrogen (%N) and the carbon stable isotope composition (δ^13^C) of the lyophilized HAs were determined with a Costech Elemental Combustion System 102 elemental analyzer (Costech Instruments) coupled with a Thermo Scientific 103 Delta V Advantage Isotope Ratio Mass Spectrometer (Thermo Scientific). Isotopic results were expressed in the usual δ notation in parts per mil (‰) versus the international standard Vienna Pee Dee Belemnite (V-PDB). Caffeine IAEA 600 was used as certified reference material.

#### UV-vis spectrometry

HA solutions were prepared by dissolving 5.0 ± 0.1 mg of each sample in 10 mL of 0.1 M phosphate buffer (pH 7.0). UV-vis spectra (200–600 nm) were recorded with a UV-visible spectrophotometer (Cary 1E, Varian), using a 1-cm quartz cuvette, after properly diluting and equilibrating the prepared solution at room temperature (20°C) for 24 h.

The E4/E6 ratios were calculated from absorbance measurements at 465 and 665 nm. The specific UV absorbance at 254 nm (SUVA254) was determined by normalizing the UV absorbance at 254 nm with respect to the C concentration in the cuvette (Shirshova et al. 2006).

#### Fourier transform infrared (FTIR) spectrometry

The FTIR spectra of freeze-dried HAs were recorded on a Perkin Elmer FTIR spectrometer (Spectrum 100) using a universal ATR sample accessory, over an interval of 4000 to 800 cm^−1^ (32 scans in each acquisition). Spectra were recorded in transmission mode but are reported in absorbance units to calculate peak intensities. A linear baseline correction was applied to compare spectra (Inbar et al. 1989). Ratios between main characteristic peaks (i.e., 3400, 1720, 1625 cm^−1^) to the 1020 cm^−1^ peak were calculated after baseline compensation.

#### Fluorescence spectrometry

Fluorescence excitation emission matrix (EEM) spectroscopy of HA solutions (diluted to 80 mg L^−1^) was recorded with an Agilent Technologies Cary Eclipse fluorescence spectrometer using quartz cuvettes (1 cm × 1 cm). Excitation and emission wavelength ranges were set from 220 to 500 nm (5-nm intervals) and from 300 to 650 nm, respectively. The scan rate was 600 nm min^−1^ (average time of 0.1 s and data interval of 1 nm). The concentration of 80 mg L^−1^ was chosen after preliminary tests (Electronic Supplementary Material, Fig. S3) according to the recommendations of Zimmermann et al. (1997).

#### Electron spin resonance (ESR)

The free radical content of HAs was measured using a Bruker EMX EPR spectrometer operating in the X-band (9.5 GHz). About 40 mg of sample (equivalent to 10 mm in height) were placed in 3.5-mm quartz tubes. Each sample was analyzed at room temperature in duplicate, and the results are reported as the number of spins per gram of C (spins g C^−1^) ± standard deviation. Other instrumental parameters were: center field 3410 G, sweep width 160 G, sweep time 60 s, microwave power 0.2 mW, modulation amplitude 1 G, and receiver gain 104. The number of scans varied from 1 to 9 as a function of the signal to noise ratio of each sample. The microwave power of 0.2 mW was chosen after performing the power saturation curve. Quantification of radicals was performed by the secondary standard method using Cr(III)MgO (g = 1.9797) as a paramagnetic marker (permanently placed in the resonance cavity) calibrated with strong pitch reference (Bruker) of known free radical content (Martin-Neto et al. 2001).

### Statistical analysis

Measurements and analyses on soils were based on oven-dried soil, replicated three times, and reported as mean ± standard deviation (SD). Data were analyzed using analysis of variance (ANOVA) with Tukey’s honest significant difference (HSD) post hoc test. Differences between treatments were considered significant at *P* < 0.05 and identified in the figures and tables with different letters. Data were statistically analyzed with R software (R Development Core Team 2010).

## Results and discussion

### Changes in soil chemical properties

Soil pH and the amount of carbonates are the main differences between the two soils compared in this study (Table 1). Soil washing increased the pH of the acidic soil significantly from 5.1 to 5.9 and had only a slight effect on the calcareous soil (from 6.7 to 7.1) due to its strong buffering capacity. Recultivation had negligible effects on either soil pH or carbonates.

**Table 1:** Soil properties and potentially toxic metals in original calcareous and acidic soil after CaEDTA soil washing (remediated) and after two cycles of cultivation (re-cultivated)

Soil type	Calcareous soil			Acidic soil		
Treatment	Original	Remediated	Re-cultivated	Original	Remediated	Re-cultivated
Sand (%)	42.2	34.8	n.d.**	42.5	31.5	n.d.
Silt (%)	47.7	55.3	n.d.	45.9	52.1	n.d.
Clay (%)	10.1	9.9	n.d.	11.6	16.4	n.d.
pH (CaCl_2_)	6.7 ± 0.1*	7.1 ± 0.2	7.2 ± 0.2	5.1 ± 0.1	5.9 ± 0.2	6.2 ± 0.2
CaCO_3_ (%)	20.3 ± 0.9	17.0 ± 1.1	16.5 ± 1.4	1 ± 0.3	1 ± 0.4	0 ± 0.3
TOC (mg g^−1^)	38.4 ± 2.8	39.8 ± 3.3	37.7 ± 2.9	26.2 ± 1.7	24.6 ± 2.1	24.4 ± 1.9
Total N (mg g^−1^)	3.7 ± 0.3	3.9 ± 0.2	3.4 ± 0.3	2.9 ± 0.2	2.7 ± 0.2	2.5 ± 0.2
CEC (cmol_+_ kg^−1^)	29.3	36.6	n.d.	16.4	20.0	n.d.
P_2_O_5_ (mg kg^−1^)	106	106	n.d.	4	7	n.d.
K_2_O (mg kg^−1^)	173	182	n.d.	207	214	n.d.
Fe (mg kg^−1^)	31,388	30,180	29,750	35,800	38,300	37,250
Mn (mg kg^−1^)	828	392	450	931	672	691
Cd (mg kg^−1^)	25.9 ± 0.3	11.7 ± 0.3	12.5 ± 0.6	4.2 ± 0.1	1.5 ± 0.1	1.4 ± 0.2
Pb (mg kg^−1^)	4029 ± 10	1695 ± 17	1620 ±1 8	774 ± 11	162 ± 6	174 ± 4
Zn (mg kg^−1^)	2219 ± 16	1905 ± 48	1901 ± 52	339 ± 5	229 ± 4	235 ± 13

*Standard deviation

**n.d., not determined

The original Pb concentrations were 4029 ± 10 and 774 ± 11 mg kg^−1^ in the calcareous and acidic soils, respectively. Both greatly exceeded the respective thresholds of the national regulation levels of 530 and 500 mg kg^−1^ for Slovenia and Austria (Official Gazette of Republic of Slovenia S 1996; Austrian Standards Institute A 2013). CaEDTA remediation removed 58% and 79% of the Pb from the calcareous and the acidic soils, respectively (Table 1). In spite of the fact that a higher CaEDTA dose was used (60 versus 100 mmol kg^−1^), Pb extractability was hindered in the calcareous soil by the abundance of Ca ions, derived from the dissociation of carbonates, which stabilized the CaEDTA complex (Manouchehri et al. 2006). The Zn content decreased from 2219 to 1905 and from 339 to 229 mg kg^−1^ in the calcareous and acidic soils, respectively, corresponding decreases of 14% and 32%, respectively. Cadmium showed a percentage decrease that was comparable to Pb. Iron was apparently not affected, while Mn decreased significantly (52% and 28% in the calcareous and acidic soils, respectively).

### Quantitative changes in soil organic matter fractions

In both soils, the total soil organic C (TOC) was not significantly changed by the soil washing process, although we found a statistically non-significant TOC decrease (−6.1%) in the acidic soil, which did not recover after recultivation (Table 2). Although washing did not affect the total amount of SOC, we found significant losses in total extractable carbon (TEC), corresponding to 24% and 26% in calcareous and acidic soils, respectively (Table 2). Although the calcareous soil had a much higher TOC content than the acidic soil (38.4 versus 26.2 mg C g^−1^), the soils yielded similar amounts of extractable C (15.6 and 13.9 mg C g^−1^) and were subject to similar TEC losses during washing. These losses occurred mainly in the free TEC fraction (TEC extracted with 0.5 M NaOH), while the bound TEC (extracted by subsequent extraction with alkaline pyrophosphate) did not decrease in the calcareous soil. In acidic soil, the bound TEC even increased, probably due to the increase in the soil pH (from 5.1 to 5.9) and the release of Ca ions by CaEDTA, which favored the binding of extractable compounds to soil minerals (Rowley et al. 2018). The total amounts of HAs decreased in calcareous and acidic soils by 23% and 36%, respectively. This decrease concerned only the free HA fractions, which decreased in calcareous and acidic soils by 45% and 53%, respectively. Similar to TEC, the bound HAs not only remained unchanged in calcareous soils, but even increased significantly in acidic soil: from 1.13 mg C g^−1^ in the original soil to 1.94 mg C g^−1^ in the CaEDTA washed soil.

**Table 2:** Soil organic C and its fractionation in calcareous and acidic soil (original) after CaEDTA soil washing (remediated) and after two cycles of cultivation (re-cultivated) expressed either as mg C g^−1^ soil and as a percentage of SOC or TEC

Soil type	Treatment	OC fraction	SOC	TEC		HA		FA		NH	
			mg C g^-1^	mg C g^−1^	% to SOC	mg C g^−1^	% to TEC	mg C g^−1^	% to TEC	mg C g^−1^	% to TEC
Calcareous	Original	Total	38.4 a*	15.16 a	39.5	9.74 a	64.2	2.67 a	17.6	2.76 a	18.2
		Free		6.67 a	17.4	4.30 a	64.5	1.02 a	15.3	1.35 a	20.2
		Bound		8.49 a	22.1	5.44 a	64.0	1.65 a	19.4	1.41 a	16.6
	Remediated	Total	39.8 a	11.57 b	29.1	7.53 b	65.1	2.15 b	18.6	1.89 b	16.4
		Free		4.00 b	10.0	2.22 b	55.6	0.69 b	17.4	1.08 b	27.0
		Bound		7.58 a	19.0	5.31 a	70.1	1.46 a	19.2	0.81 c	10.7
	Re-cultivated	Total	37.7 a	11.76 b	32.9	7.64 b	64.9	2.07 b	17.6	2.05 b	17.4
		Free		3.60 b	10.1	1.99 b	55.4	0.71 b	19.7	0.90 b	25.0
		Bound		8.16 a	22.9	5.64 a	69.2	1.37 a	16.7	1.15 b	14.1
Acidic	Original	Total	26.2 a	13.92 a	53.1	8.65 a	62.1	2.14 a	15.3	3.14 a	22.6
		Free		11.63 a	44.4	7.51 a	64.6	1.71 a	14.7	2.42 a	20.8
		Bound		2.29 a	8.7	1.13 a	49.6	0.43 a	18.8	0.72 a	31.6
	Remediated	Total	24.6 a	10.25 b	41.7	5.48 b	53.5	1.93 a	18.8	2.84 a	27.7
		Free		6.70 b	27.2	3.55 b	52.9	1.16 b	17.3	2.00 a	29.8
		Bound		3.55 c	14.4	1.94 b	54.7	0.77 b	21.6	0.84 a	23.7
	Re-cultivated	Total	24.4 a	8.74 b	35.8	4.34 b	49.7	1.69 a	19.3	2.71 a	31.0
		Free		5.79 b	23.7	2.67 b	46.2	1.13 b	19.6	1.98 a	34.2
		Bound		2.96 b	12.1	1.67 b	56.5	0.56 a	18.8	0.73 a	24.8

*SOC*, soil organic C; *TEC*, total extractable C; *HA*, humic acids; *FA*, fulvic acids; *NH*, non-humic C

*Different letters refer to statistical differences (Tukey’s HSD post hoc test, *P* < 0.05); comparisons are exclusively between the same fraction of the three treatments: original, remediated, and re-cultivated and of the same soil

Fulvic acids (FAs) showed a decrease in both soils that paralleled the TEC findings, with no significant changes observed in percentage terms (Table 2). The loss of FAs in the calcareous soil was also largely due to the free FA fraction, which showed a significant decrease in the acidic soil that was concomitant with a slight but statistically significant increase in the bound FA fraction.

The non-humic fraction (NH) of extractable organic matter represented about 10 to 27% and 21 to 31% of the free and bound TEC fractions of the calcareous and acidic soils, respectively. The remediation treatment in the calcareous soil caused a C loss in this fraction proportional to that of TEC and occurred in both the free and bound fractions. No significant changes were observed in the acidic soil (Table 2). Non-humified organic matter (NH) is important in soil fertility, as it is the main fraction that directly supports the soil microbial biomass in its growth, survival, and activities (De Nobili et al. 2001). The limited effect of soil washing on this SOM fraction, in particular in the acidic soil, is in agreement with the results obtained by Kaurin et al. (2015, 2018, 2020), which showed preservation of microbial and enzyme activities after CaEDTA soil washing.

Looking at the qualitative composition of TEC, non-humified and humified C, the increase in the NH/(FA + HA) ratio clearly shows that soil washing in both soils caused the preferred loss of the free fractions of humified organic C (2.08 and 3.96 mg g^−1^ HAs and 0.33 and 0.55 mg g^−1^ FAs from the calcareous and acidic soils, respectively), while the bound TEC fractions showed the opposite trend, with a greater loss of NH than humified C (Electronic Supplementary Material, Table S1). Before remediation, the NH/(FA + HA) ratio was 0.22 and in the calcareous soil did not change after soil washing and recultivation. The NH/(FA + HA) ratio was slightly higher in the acidic soil (0.29), indicating a lower degree of SOM humification, and further increased to 0.38 after soil washing and 0.45 after recultivation. The remediation treatment therefore had a stronger influence on the humified fractions (FAs and HAs) of the acidic soil. Recultivation of remediated soils with two series of vegetable crops did not cause significant quantitative changes in the TEC, HAs, and FAs in the calcareous soil but increased the bound NH fraction (Table 2). This increase did not compensate for the losses in the free fraction so the NH/(FA + HA) ratio of TEC remained practically unchanged. However, recultivation restored the original ratios of free/bound NH in both soils (Electronic Supplementary Material, Table S1).

The ratios of free to bound extractable C (TEC) ranged from 0.79 in calcareous soil to 5.08 in acidic soil, in accordance with the much higher amount of binding polycations (especially Ca and Mg) present in calcareous soil (Electronic Supplementary Material, Table S1). In both soils, CaEDTA soil washing caused this ratio to decrease, confirming a greater loss of the free fraction. In particular, while this fraction was lost from the calcareous soil, in the acidic soil part of the free extractable C was bound to soil minerals, which led to an increase in the bound TEC fraction.

Indeed, the free humified fractions have well-documented stimulating effects on plant growth (Chen et al. 2004; Vujinović et al. 2020) and play an important role in plant nutrition as complexing agents of several micronutrients, including Fe and Mn (Cesco et al. 2000). Free HAs also represent a slowly degradable N reserve (Stevenson 1994), while the degradation of bound HAs is hindered not only by intrinsic chemical stability but also by binding to minerals.

The ratios of free/bound humic substances (HA + FA) decreased from 0.75 to 0.43 and from 5.9 to 1.7 in calcareous and acidic soils, respectively, which illustrates the stronger influence of CaEDTA soil washing on the free humic substances fraction in acidic soils (Electronic Supplementary Material, Table S1). Yip et al. (2010) showed that the adsorption of HAs on the surface of soil minerals after EDDS washing reached up to 75%, and the same effect can be assumed for CaEDTA. The simultaneous increases in pH and the Ca ion concentration are probably the most important factors responsible for the observed shift from free to bound humic substances.

The most important fractions of the SOM in terms of stability and permanent effect on the physical properties of soils are the bound HA and FA fractions. These fractions were preserved by washing the soil with CaEDTA, whereas the free humified fractions (FA + HA) decreased significantly. In addition to the targeted PTMs, EDTA forms stable complexes with a variety of polycations in soils such as Al, Ca, Fe, and Mg (Zeng et al. 2005), and this may have an adverse effect on the structure and physical properties of the soil matrix. Similarly, Tsang et al. (2007) observed the dissolution of native Fe/Al oxides, Ca carbonates, and organic matter in soils that have been flushed with Na_2_EDTA. It was reported that CaEDTA, as used in this study, has a less negative effect on soil properties (Lestan 2017; Theodoratos et al. 2000). Indeed, the soil washing solution provides Ca^2+^ ions that effectively promote the formation of cationic bridges. In this situation, the binding of organic matter to soil minerals is maintained, and this is the main reason why the bound humic fractions have not been reduced like the free humic fractions.

### Qualitative changes in the HA fractions

#### The C/N ratio and carbon isotope composition of HAs

**Fig. 2:**
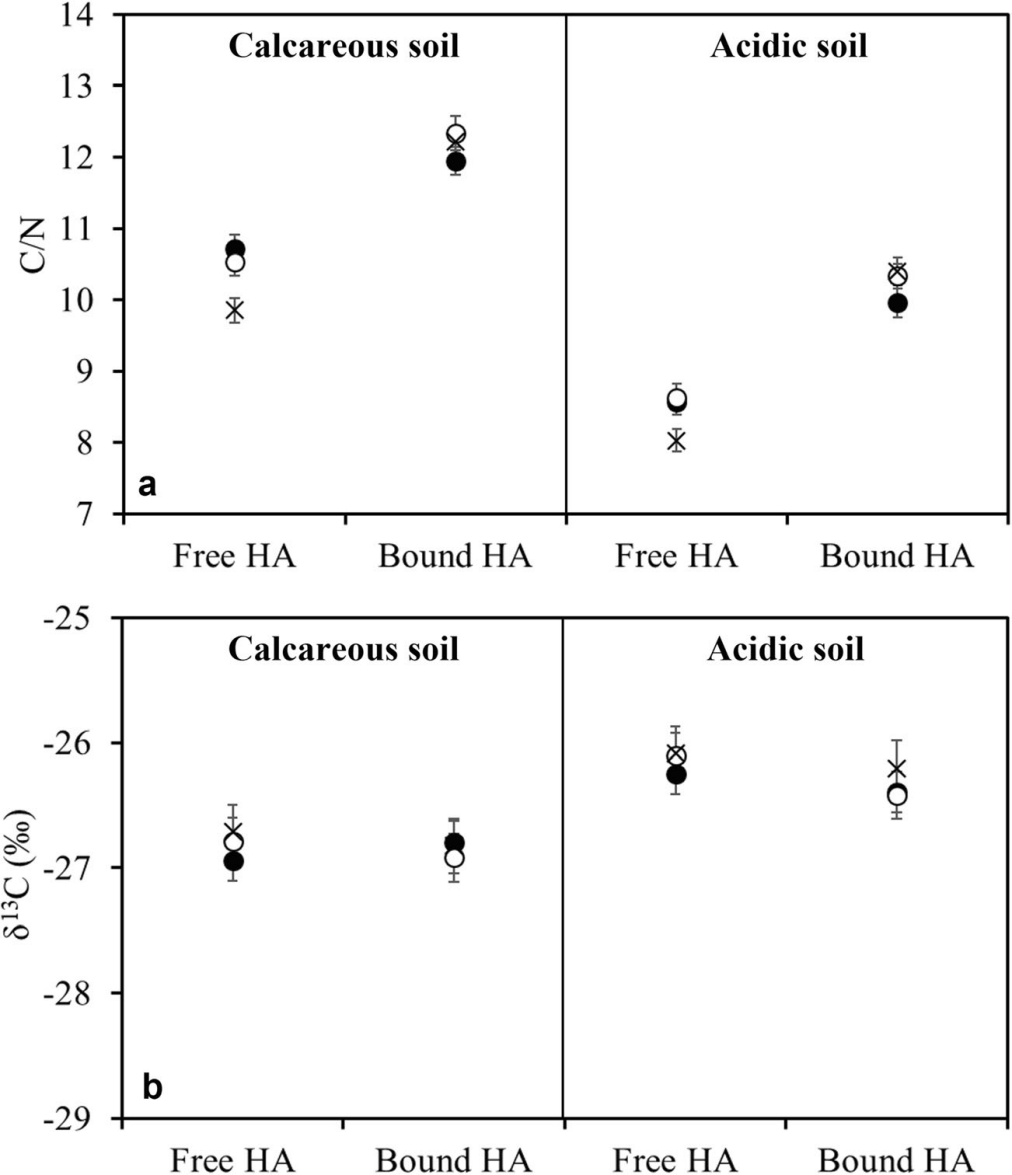
C/N ratio (**a**) and carbon stable isotope composition (**b**) of free and bound humic acids (HAs) extracted from the original (●), remediated (o), and re-cultivated (x) soil samples. Bars represent the standard deviation of the mean (*n* = 3)

The C/N ratios of free and bound HAs extracted from the calcareous soil were on average about 20% higher than from the corresponding fraction in acidic soil (Fig. 2a). On the other hand, in both soils, the C/N ratios of bound HAs were significantly higher than those of free HAs, indicating a proportionally lower N content. Soil washing and recultivation did not influence the C/N ratio of bound HAs, while recultivation significantly decreased the C/N ratio of free HAs in both soils. This suggests the incorporation of new nitrogenous components in the free HAs by plant growth.

A difference of about −1‰ in the δ^13^C values was found between the corresponding fractions extracted from calcareous and acidic soils due to the different origins of their organic C inputs. The carbon stable isotopic composition of both the free and bound HAs did not allow the different soil treatments to be distinguished (Fig. 2b), indicating that CaEDTA soil washing did not cause significant isotopic fractionation. Also, recultivation did not affect the ^13^C signature of HAs: this was expected, as HAs are the most stable components of soil organic matter, and their formation would eventually be accompanied by an increase in SOC, which we did not observe.

#### UV-vis spectroscopy of HAs

The specific absorbance (SA) spectra of free and bound HAs (Electronic Supplementary Material, Fig. S1) increased monotonically with decreasing wavelength and showed a shoulder around 260–280 nm (more pronounced in the free HAs). Each spectrum represents the sum of the absorption bands of the component chromophores (Sparks et al. 1996), typically C=C and C=O groups (Stevenson 1982).

Differences between spectra have been highlighted by taking into account the spectral parameters E4/E6 and SUVA254. It is often claimed that the E4/E6 ratio is related to the aromaticity and degree of condensation of aromatic carbons in the HAs and has been proposed as a humification index (Stevenson 1982; Zalba et al. 2016). This ratio is independent of the concentration of HAs but varies according to their type and origin (Tahiri et al. 2016). However, Chen et al. (1977) showed that the E4/E6 ratio increases when the molecular weight of the humic substances decreases. Changes in the E4/E6 ratios were negligible for the bound HA fraction after remediation (Table 3). In contrast, a significant increase in this ratio in the free HAs of both soils was observed after soil washing with CaEDTA. This indicates that the loss of HAs (Table 2) was either associated with the removal of larger molecular components from this fraction during the washing treatment (which is unlikely, due to its lower solubility) or, more likely, was associated with an interruption of the intermolecular bonds. Indeed, CaEDTA (due to its relatively high concentration and complexing capacity) is able to disrupt cationic bridges between HA molecules, resulting in an apparent decrease in molecular size. This interpretation is confirmed by the fact that SUVA254 values, which estimate the abundance of UV-absorbing chromophores (typically aromatic and carboxylic electron systems and their conjugates; McDonald et al. 2004), remained practically constant in all HA fractions of both soils. This indicated that neither the remediation treatment nor the recultivation caused significant changes in intrinsic structural complexity or aromaticity and that the increase in the E4/E6 ratio was due to the disruption of polycationic bridges between the HA molecules.

**Table 3:** UV-vis spectroscopic parameters of free and bound HA. Different letters refer to statistical differences (*P* < 0.05) between the same fraction of the three treatments

Soil type	Treatment	E_4_/E_6_		SUVA_254_	
		Free HA	Bound HA	Free HA	Bound HA
Calcareous	Original	5.8 a	6.0 a	4.5 a	5.2 a
	Remediated	7.1 b	5.9 a	4.3 a	5.3 a
	Re-cultivated	6.8 b	5.7 a	4.5 a	5.1 a
Acidic	Original	4.9 a	5.3 a	4.1 a	4.3 a
	Remediated	6.3 b	5.6 a	3.8 a	4.5 a
	Re-cultivated	6.2 b	5.4 a	3.9 a	4.6 a

#### FTIR characterization of HAs

FTIR spectra reflect the predominance of oxygen-containing functional groups (i.e., C-OH, OH, and C=O) in humic substances (Schnitzer 1999). All FTIR spectra (Electronic Supplementary Material, Fig. S2) showed the typical absorption bands of HAs (Giovanela et al. 2004) but allowed the distinction between free and bound HA fractions, confirming that these fractions correspond to different types of molecules. Bound HAs showed broader and less resolved bands than free HAs, indicating a larger molecular complexity and a stronger contribution of intramolecular/intermolecular H-bonds (Bravo et al. 2020).

The intensity ratios of specific characteristic bands (Table 4) allowed highlighting of differences in the composition of HAs after soil washing and recultivation. Indeed, FTIR spectra of free HAs extracted from the remediated acidic soil showed an increase in absorption at 3400 cm^−1^ (phenolic O-H stretching) and at 1625 cm^−1^ (aromatic C=C, functional quinone groups) in relation to absorption at 1020 cm^−1^ (carbohydrate C-O and C-C stretching). The same was observed for free HAs in calcareous soil but with less variation in aromatic structures and a greater increase in aliphatic parts. The 1020-cm^−1^ band can be attributed to the C-O and C-C stretching vibrations of carbohydrate rings, and its relative decrease indicated a lower contribution of this type of structure in the remediated free HAs of both soils. Minor changes were observed in the bound HAs after remediation, which are related to the fact that this fraction did not undergo any quantitative change and brought about a slight increase in both aliphatic and aromatic structures (C-H stretching and O-H stretching and bending of phenols, aromatic C=C stretching) in the acidic soil. In calcareous soil, the structural composition of the bound HAs underwent virtually no modification with soil washing, and no further modification occurred with recultivation.

**Table 4:** FTIR spectroscopic of free and bound HA in calcareous and acidic soils (original) after CaEDTA soil washing (remediated) and after two cycles of cultivation (re-cultivated): the ratio between principal peaks. Different letters refer to statistical differences (*P* < 0.05) between the same fraction of the three treatments

Soil type	Treatment	3400/1020 cm^−1^		1720/1020 cm^−1^		1625/1020 cm^−1^		1215/1020 cm^−1^	
		Free HA	Bound HA	Free HA	Bound HA	Free HA	Bound HA	Free HA	Bound HA
Calcareous	Original	0.18 a	0.25 a	0.24 a	0.46 a	0.34 a	0.58 a	0.29 a	0.49 a
	Remediated	0.27 a	0.27 a	0.34 b	0.46 a	0.61 b	0.66 a	0.65 b	0.61 a
	Re-cultivated	0.31 b	0.32 a	0.34 b	0.51 a	0.65 b	0.69 a	0.63 b	0.64 a
Acidic	Original	0.19 a	0.25 a	0.48 a	0.31 a	0.49 a	0.48 a	0.48 a	0.28 a
	Remediated	0.30 b	0.26 a	0.60 b	0.39 a	0.63 b	0.58 a	0.52 a	0.47 b
	Re-cultivated	0.30 b	0.23 a	0.59 b	0.38 a	0.63 b	0.53 a	0.48 a	0.42 b

### EEM fluorescence of HAs

**Fig. 3:**
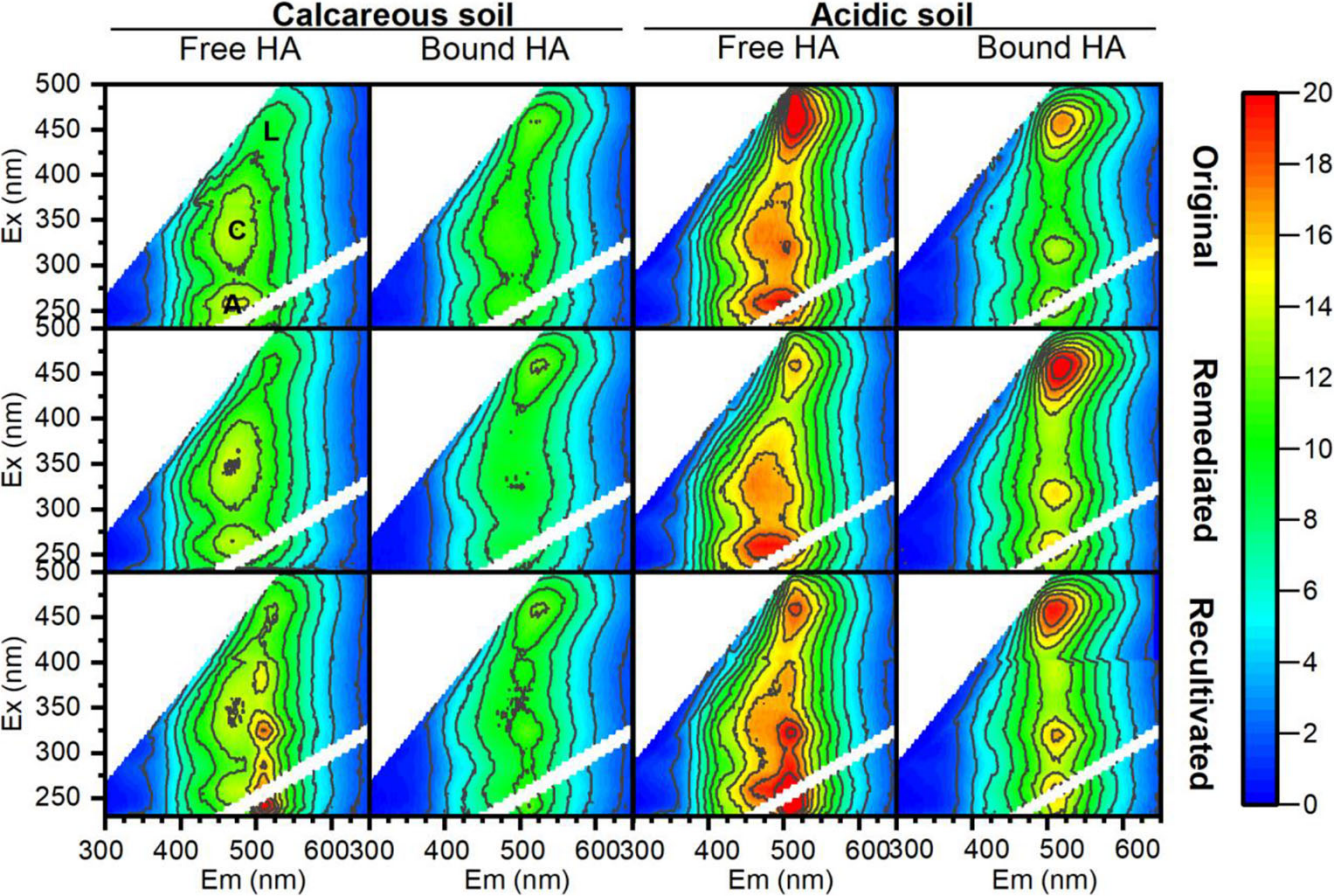
EEM fluorescence spectra of free and bound HAs in original, remediated, and re-cultivated calcareous and acid soils

EEM fluorescence spectroscopy has allowed the identification of different fluorescent peaks in HAs derived from a number of origins (Santos et al. 2015). Figure 3 shows the EEM fluorescence spectra of free and bound HA extracts from both calcareous and acidic soils. All spectra were characterized by the presence of three main peaks, in good agreement with those reported for other soil HAs (Antízar-Ladislao et al. 2006; Enev et al. 2015). The principal peak (λem/λex = 455/510 nm), which occurred at long excitation and emission wavelengths, is characteristic of HAs derived from lignin (peak L) and other complex aromatic macromolecules (Gao et al. 2019). Peak C (λem/ λex = 310/500 nm), which indicates phenol-like or naphthol-like structures (Halim et al. 2013), is also typical for humic substances (Coble 1996). Finally, a peak at low excitation wavelengths (λem/λex = 260/500 nm) is typical for HAs from terrestrial environments (peak A) (Tadini et al. 2017).

All HA fractions extracted from the acidic soil showed a much higher fluorescence intensity than fractions extracted from calcareous soil, and large differences were found for the free HA fractions. While there was no significant difference between the bound HA fractions, recultivation caused a redshift of about 20 nm and an increase in the intensity of peaks A and C in the free HAs, indicating that some new fluorophores were incorporated in the HA fraction in the presence of plants. Recultivation caused the incorporation of new nitrogenous components in the free HAs (Fig. 3). This is consistent with the hypothesis that free HAs are a younger and more dynamic fraction than bound HAs.

#### ESR of HAs

A representative solid-state ESR spectrum is shown in the Electronic Supplementary Material, Fig. S4. Each spectrum is composed of the signal of the organic radicals present in the sample and the signal of the secondary standard. All spectra were characterized with a g value of 2.004 ± 0.001, which corresponds to oxygen-centered radical species such as semiquinones and methoxybenzenes (Martin-Neto et al. 1998; Watanabe et al. 2005). The concentration of free radicals in the extracted HAs is given in Table 5. Compared to free HAs, a significantly higher concentration of radicals was found in the corresponding bound HAs, in accordance with their stronger aromatic character, which is confirmed by their SUVA254 values and FTIR spectra. The line width of the radical signals was smaller in bound HAs than free HAs, suggesting a more stable molecular structure, with a longer lifetime of the radical species in the bound HAs (Watanabe et al. 2005). Soil washing with CaEDTA had no effect on the concentration of free radicals either of the HA fractions in the calcareous soil (Table 5), while in the free HAs in the acidic soil, a small but significant increase was observed, probably due to the increase in the soil’s pH. Changes in the free radical content of the bound HA fraction in the acidic soil were not significant.

**Table 5:** The free radical concentration in the extracted free and bound HA. Different letters refer to statistical differences (*P* < 0.05) between the same fraction of the three treatments

Soil type	Treatment	Spins g C^−1^ (*10^17^)		Line width (G)	
		Free HA	Bound HA	Free HA	Bound HA
Calcareous	Original	2.78 a	5.24 a	7.32 a	5.63 a
	Remediated	2.65 a	5.40 a	7.44 a	5.56 a
	Re-cultivated	2.82 a	5.53 a	7.55 a	5.64 a
Acidic	Original	2.50 a	4.48 a	7.80 a	5.74 a
	Remediated	2.91 b	4.63 a	8.00 a	5.64 a
	Re-cultivated	2.89 b	4.80 a	7.86 a	5.78 a

## Conclusions

Soil washing with CaEDTA resulted in a selective reduction in the FAs and HAs which are not bound to soil minerals (free FAs and HAs) but preserved most of the bound fractions, which are linked by cationic bridges to the surface of soil minerals. It can therefore be assumed that the surface properties of the solid matrix of both soils were not damaged by the washing treatment. Surprisingly, no large difference has been found between the two soils examined, irrespective of their contrasting mineralogy and pH.

Recultivation with two cycles of crops was not able to restore, either qualitatively or quantitatively, the most dynamic fractions of the SOM, such as the NH and free HA fractions. In order to support soil fertility after CaEDTA soil washing, we therefore propose to use organic amendments rich in humified organic matter, such as mature farmyard manure, compost, or peat, as a way to rapidly restore the free fraction of the humified SOM.

## Supplementary Information

ESM 1 (DOCX 509 kb)
