# Supplementary material for: Changes in organic matter composition caused by EDTA washing of two soils contaminated with toxic metals
Source: Environ Sci Pollut Res Int. 2021 Jul 28;28(46):65687–99. doi: 10.1007/s11356-021-15406-z (PMC8636399; doi:10.1007/s11356-021-15406-z)
Supplement: Supplementary file 1 — (DOCX 509 kb) [file 11356_2021_15406_MOESM1_ESM.docx]

**Changes in organic matter composition caused by EDTA washing of two soils contaminated with toxic metals**

Erika Jez^a^, Carlo Bravo^b^, Domen Lestan^c^, Simon Gluhar^c^, Ladislau Martin-Neto^d^, Maria De Nobili^b^ and Marco Contin^b*^

**Supplementary material**


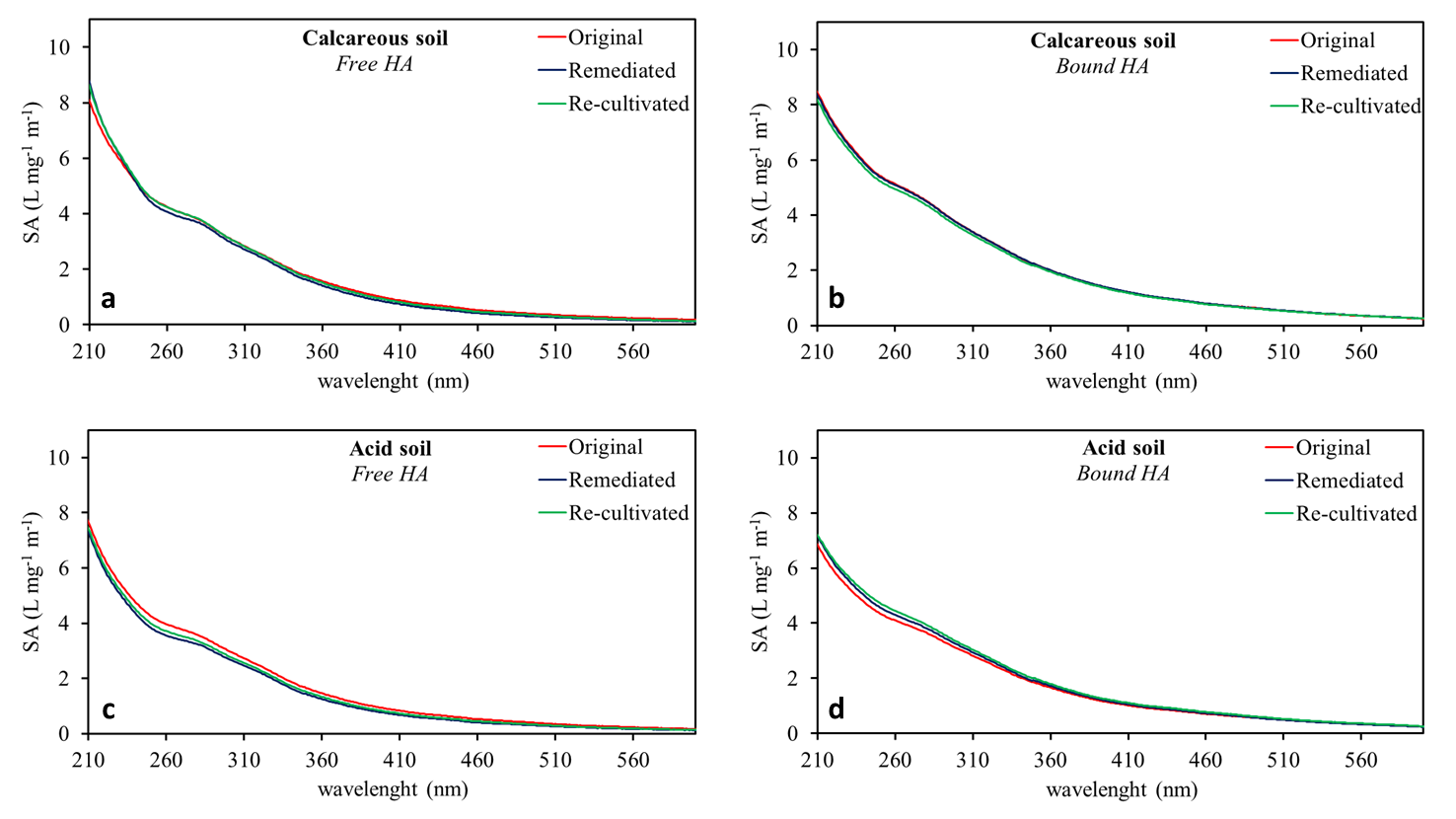


**Fig. S1** Specific absorbance (SA) UV-vis spectra of free and bound HA in calcareous and acidic soil (Original), after CaEDTA-soil washing (Remediated) and after two cycles of cultivation (Re-cultivated).


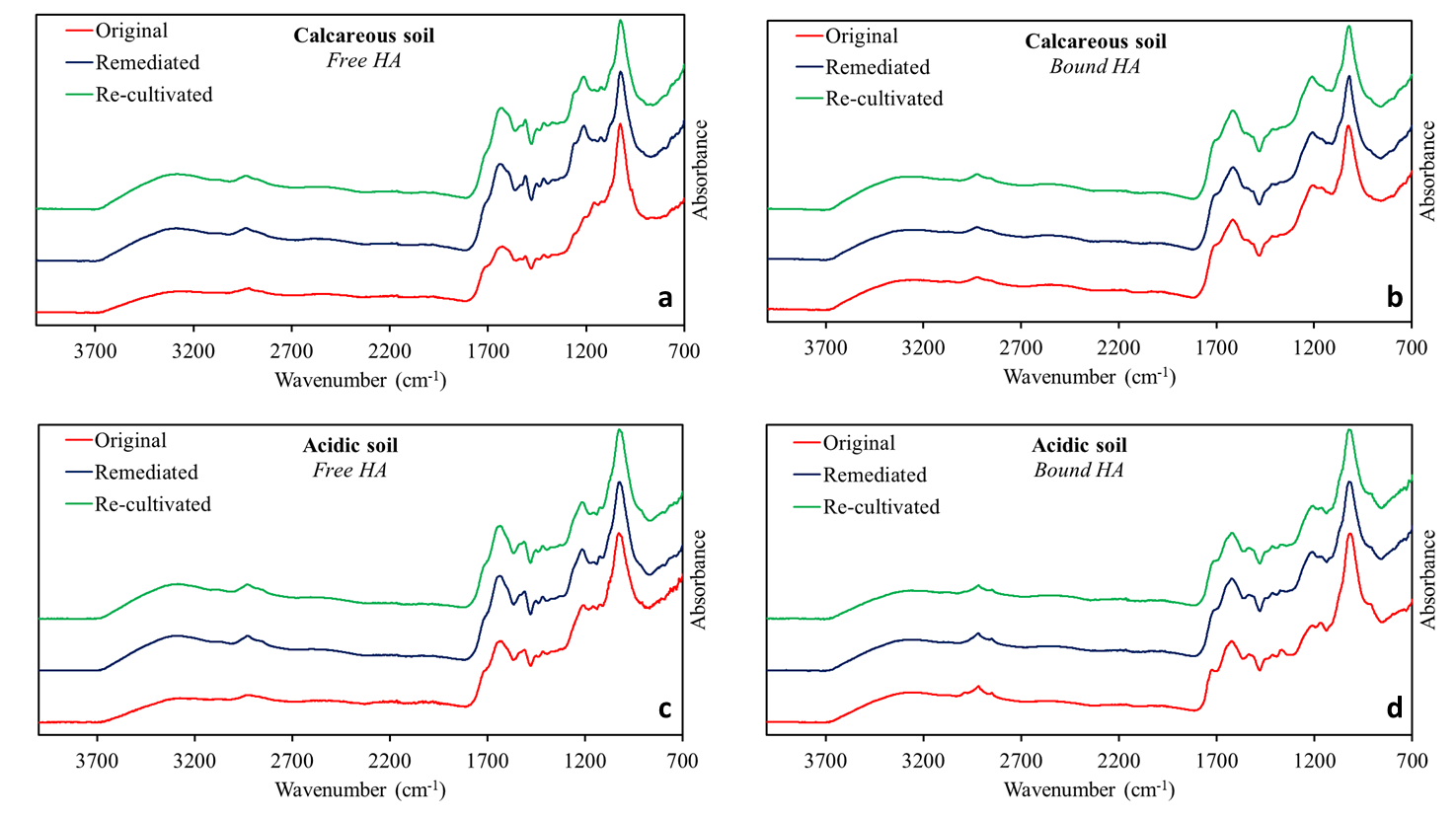


**Fig. S2** FT-IR spectra of free and bound HA in calcareous and acidic soil (Original), after CaEDTA-soil washing (Remediated) and after two cycles of cultivation (Re-cultivated).


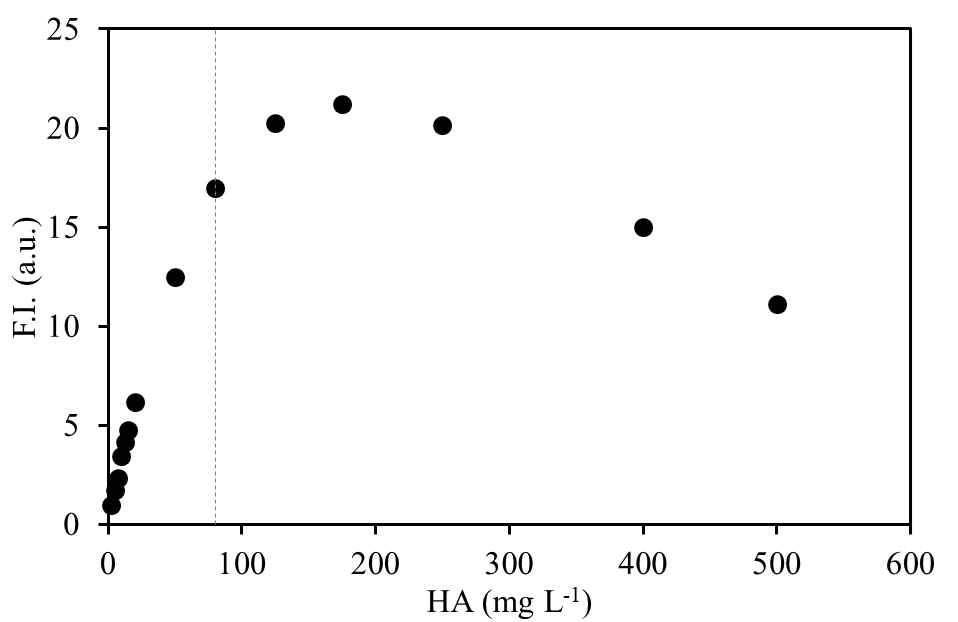


**Fig. S3** Determination of the optimal concentration of HA solution for the fluorescence excitation emission matrix (EEM) measurements. The picture shows the fluorescence intensities of the peak at excitation/emission wavelengths of 440/510 nm of one representative HA sample (acidic soil, original) as function of the HA concentration in the quartz cell. The concentration of 80 mg L^-1^ (dotted grey line) was chosen for all fluorescence measurements.


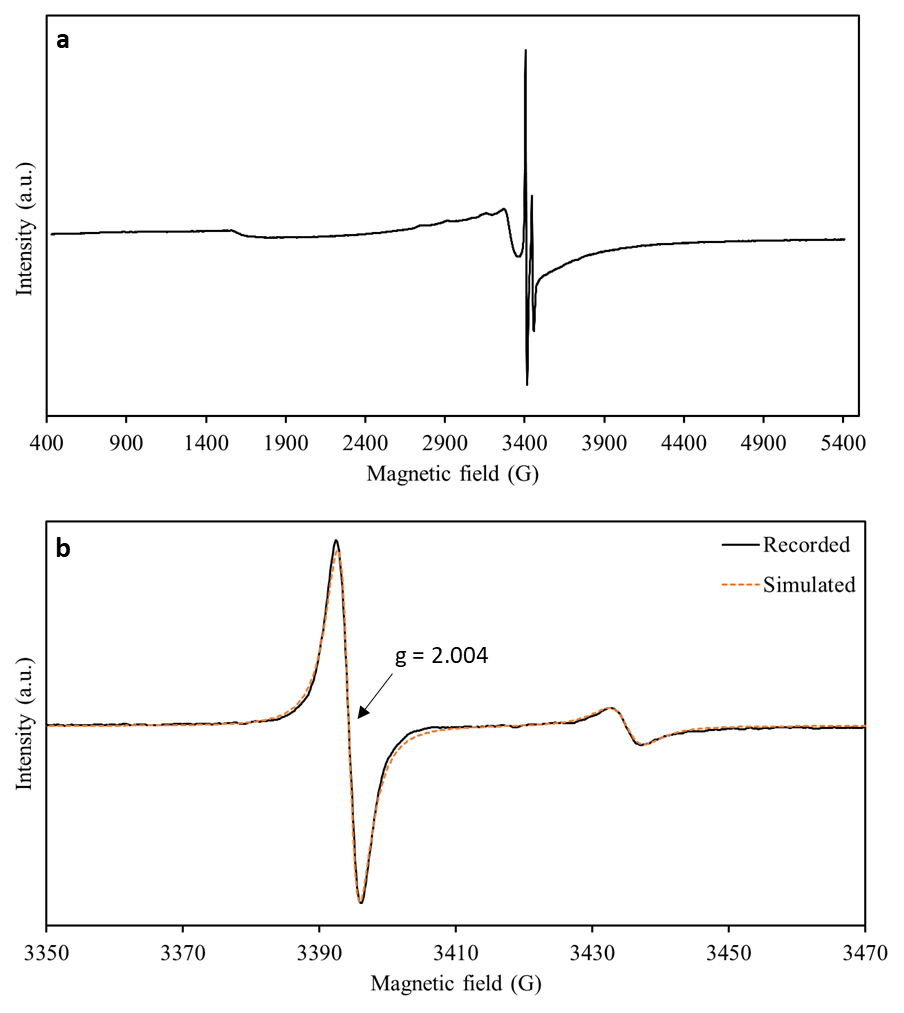


**Fig. S4** ESR spectrum of a representative HA sample (Free HA of remediated acid soil) (a). Window from 3350 to 3470 G of the same EPR spectrum where the free organic radicals and the secondary standard signal are expanded (b). Black solid line: recorded spectrum; Orange dashed line: simulated spectrum.

**Table S1** Non humic (NH) to humic (FA+HA) ratio and free to bound ratio in calcareous and acidic soils (Original), after CaEDTA-soil washing (Remediated) and after two cycles of cultivation (Re-cultivated).

| Soil type | Treatment | NH/(FA+HA) | | |  | Free/Bound | | | | |
| --- | --- | --- | --- | --- | --- | --- | --- | --- | --- | --- |
|  |  | Total | Free | Bound |  | TEC | HA | FA | FA+HA | NH |
| Calcareous | Original | 0.22 a | 0.25 a | 0.20 a |  | 0.79 a | 0.79 a | 0.62 a | 0.75 a | 0.95 a |
|  | Remediated | 0.20 a | 0.37 b | 0.12 c |  | 0.53 b | 0.42 b | 0.48 a | 0.43 b | 1.33 b |
|  | Re-cultivated | 0.21 a | 0.33 b | 0.16 b |  | 0.44 b | 0.35 b | 0.52 a | 0.38 b | 0.78 a |
| Acidic | Original | 0.29 a | 0.26 a | 0.46 a |  | 5.08 a | 6.62 a | 3.96 a | 5.89 a | 3.34 a |
|  | Remediated | 0.38 b | 0.42 b | 0.31 b |  | 1.89 b | 1.83 b | 1.51 b | 1.74 b | 2.38 b |
|  | Re-cultivated | 0.45 b | 0.52 b | 0.33 b |  | 1.96 b | 1.60 b | 2.04 b | 1.71 b | 2.70 ab |

Different letters refer to statistically differences (Tukey HSD Post-hoc test P<0.05); comparisons are exclusively between the same fraction of the three treatments: original, remediated, re-cultivated, and of the same soil.
